# Genetic Variations in Neuroinflammatory Chemokine Receptor Genes in Patients with Epilepsy: An Exploratory Study

**DOI:** 10.3390/ijms27219471

**Published:** 2026-10-23

**Authors:** Kubra Cigdem Pekkoc-Uyanik, Erhan Rasit Agay, Hafize Uzun

**Affiliations:** 1 Department of Medical Biology, Faculty of Medicine, Haliç University, 34060 Istanbul, Turkey; kubrapekkoc@halic.edu.tr; 2 Sinan Sipahi Family Health Centre, Republic of Turkey Ministry of Health, 34295 Istanbul, Turkey; erhanagay@gmail.com; 3 Department of Medical Biochemistry, Faculty of Medicine, Istanbul Atlas University, 34408 Istanbul, Turkey

**Keywords:** *CCR5*, *CXCR4*, gene sequencing, polymorphism, haplotype analysis, linkage disequilibrium

## Abstract

Epilepsy is a common neurological disorder in which genetic and neuroinflammatory mechanisms may contribute to differences in seizure control. Chemokine receptor signaling plays an important role in inflammatory processes, and genetic variations in neuroinflammatory chemokine receptor genes may potentially influence the prognosis of epilepsy. This study aimed to investigate genetic variations in the neuroinflammatory chemokine receptor genes *CCR5* and *CXCR4* in patients with epilepsy and to explore their potential association with seizure control during treatment. Targeted next-generation sequencing (tNGS) was performed to investigate genetic variations in the *CCR5* and *CXCR4* genes using genomic DNA obtained from 22 patients with epilepsy receiving antiseizure medication (ASM) treatment with levetiracetam and/or valproic acid. Patients were classified into two groups according to seizure frequency to evaluate the association between genetic variation and clinical prognosis. A total of seven genetic variants were identified in the *CCR5* and *CXCR4* genes, including three variants in *CCR5* and four variants in *CXCR4*. However, sufficient genotype variation for group-wise comparisons was available only for CCR5 rs1799988 and rs1800023. No significant differences were observed in the genotype or allele distributions of the *CCR5* and *CXCR4* gene variants between the responsive and persistent groups. The two *CCR5* variants, rs1799988 and rs1800023, showed strong linkage disequilibrium (D′ = 1.000, r^2^ = 0.913) and were included in a single haplotype block. None of the identified *CCR5* haplotypes was significantly associated with seizure control. In this preliminary cohort, no statistically significant associations were detected between the analyzed genetic variations and seizure control during treatment; however, the limited sample size may have reduced the statistical power to detect potential associations. Strong linkage disequilibrium was observed between rs1799988 and rs1800023 (D′ = 1.000, r^2^ = 0.913), consistent with previously reported patterns. Given the small sample size and the limited number of informative variants, these findings should be interpreted as preliminary and hypothesis-generating rather than as evidence for or against a genetic contribution to seizure control. Larger, independent cohorts are needed to assess the potential clinical relevance of *CCR5* and *CXCR4* variation in epilepsy.

## 1. Introduction

Epilepsy is one of the most common serious neurological disorders, affecting more than 70 million people worldwide [1,2]. Epilepsy is a heterogeneous neurological disorder characterized by recurrent unprovoked seizures and diverse clinical manifestations [3]. Despite substantial advances in antiseizure medication (ASM) therapy, achieving sustained seizure control remains challenging in a proportion of patients. Although ASMs provide adequate seizure control for many individuals, a considerable subset continues to experience seizures despite appropriate treatment. This variability in treatment response reflects the complex and multifactorial nature of epilepsy and may arise from differences in genetic background, disease characteristics, drug disposition, and underlying biological pathways [4]. Understanding the factors contributing to interindividual variability in treatment response is therefore important for improving the management of patients with epilepsy and identifying potential molecular determinants of treatment outcome.

Genetic factors are increasingly recognized as potential contributors to the interindividual variability observed in epilepsy and treatment outcomes. Genetic variation may influence susceptibility to seizures, disease phenotype, and the clinical response to antiseizure medications through effects on biological pathways involved in neuronal excitability, immune regulation, and drug action [4,5]. Although several genetic variants have been investigated in relation to antiseizure medication response, the available evidence remains heterogeneous and only a limited number of genetic markers have demonstrated consistent clinical relevance [4,5]. Therefore, identifying additional genetic determinants of treatment response may provide further insight into the biological mechanisms underlying variability in epilepsy outcomes.

Neuroinflammation has increasingly been recognized as an important component of the pathophysiology of epilepsy [6]. Evidence from both experimental and human studies indicates that seizures can activate inflammatory processes in the brain, including microglial and astrocytic activation, disruption of the blood–brain barrier, and increased production of proinflammatory cytokines and chemokines [6,7]. These inflammatory mediators can influence neuronal excitability and may contribute to a lower seizure threshold and the persistence of epileptic activity. Thus, neuroinflammatory pathways may represent important biological mechanisms underlying the clinical heterogeneity of epilepsy and may contribute to differences in seizure control among individuals.

Among the mediators involved in neuroinflammatory signaling, chemokines and their receptors have attracted increasing attention in epilepsy. Chemokines regulate immune-cell migration and communication within the central nervous system and may influence inflammatory responses following seizure activity [7]. In particular, chemokine receptor signaling has been implicated in the recruitment and activation of inflammatory cells and in the modulation of neuroinflammatory processes associated with neuronal injury [8]. CCR5 is expressed in the central nervous system and has been implicated in microglial activation, leukocyte recruitment, and seizure-associated neuroinflammation [8]. Experimental studies have further suggested that modulation of the CCL5/CCR5 axis can influence neuroinflammatory responses and neuronal injury following seizures [9,10]. The CXCR4 receptor, another important chemokine receptor involved in immune-cell trafficking and inflammatory signaling, may likewise participate in neuroinflammatory processes within the central nervous system [10,11]. Together, these findings suggest that chemokine receptor signaling may represent a biologically plausible pathway through which genetic variation could contribute to interindividual differences in epilepsy.

Genetic variation in chemokine receptor genes may contribute to interindividual differences in immune signaling and inflammatory responses by altering receptor expression, ligand interaction, or downstream signaling activity [12]. In this context, polymorphisms in *CCR5* and *CXCR4* represent potential candidate variants for investigating the genetic basis of variability in epilepsy. Although alterations in chemokine and chemokine receptor signaling have been reported in epileptic brain tissue and experimental models, evidence regarding the contribution of inherited variation in *CCR5* and *CXCR4* to clinical heterogeneity in epilepsy remains limited [6,7]. In particular, the potential relationship between genetic variation in these receptors and seizure control during treatment has not been sufficiently investigated. Therefore, examining *CCR5* and *CXCR4* genetic variation may provide preliminary insight into whether neuroinflammatory signaling pathways contribute to interindividual differences in epilepsy outcomes.

Therefore, the present study aimed to investigate genetic variations in the neuroinflammatory chemokine receptor genes *CCR5* and *CXCR4* in patients with epilepsy. As a secondary exploratory objective, we evaluated whether the identified genetic variations were associated with seizure control during treatment.

## 2. Results

### 2.1. CCR5 and CXCR4 Gene Variant Analysis

Targeted sequencing of the *CCR5* and *CXCR4* genes was performed in 22 patients with epilepsy who were receiving antiepileptic treatment with levetiracetam and/or valproic acid. A total of seven variants were identified across the two genes. Three variants were detected in *CCR5* (rs1799988, rs1800023, and rs333), while four variants were identified in *CXCR4* (rs2680880, rs2471859, rs139877430, and rs2228014) (Table 1).

In the *CCR5* gene, the rs1799988 variant was detected in 17 patients, including 11 heterozygous and 6 homozygous individuals. The rs1800023 variant was identified in 16 patients, of whom 10 were heterozygous and 6 were homozygous. Both variants were upstream gene variants and classified as having a modifier effect. In addition, the rs333 variant was detected in two patients in a heterozygous. This variant corresponds to a c.554_585del deletion resulting in a p.Ser185IlefsTer32 frameshift alteration and was annotated as a high-impact variant.

In the *CXCR4* gene, the most frequently observed variant was rs2680880:A > T, which was detected in seven patients in a heterozygous. This variant was annotated as c.16-67T > A and classified as an intronic variant. The rs2471859:A > G variant was detected in three patients, whereas rs139877430:G > A was identified in two patients; both variants were located within intronic regions. The rs2228014:G > A variant was detected in one patient in the heterozygous state. This variant corresponds to c.414C > T (p.Ile138=) and was annotated as a synonymous variant.

The identified variants were predominantly located in intronic or regulatory regions of the *CCR5* and *CXCR4* genes. Among the detected variants, *CCR5* rs333 was notable because of its predicted frameshift effect and high-impact annotation. Although rs333 was annotated as a high-impact frameshift variant based on its predicted molecular consequence, its clinical significance was classified as benign.

**Figure 1:**
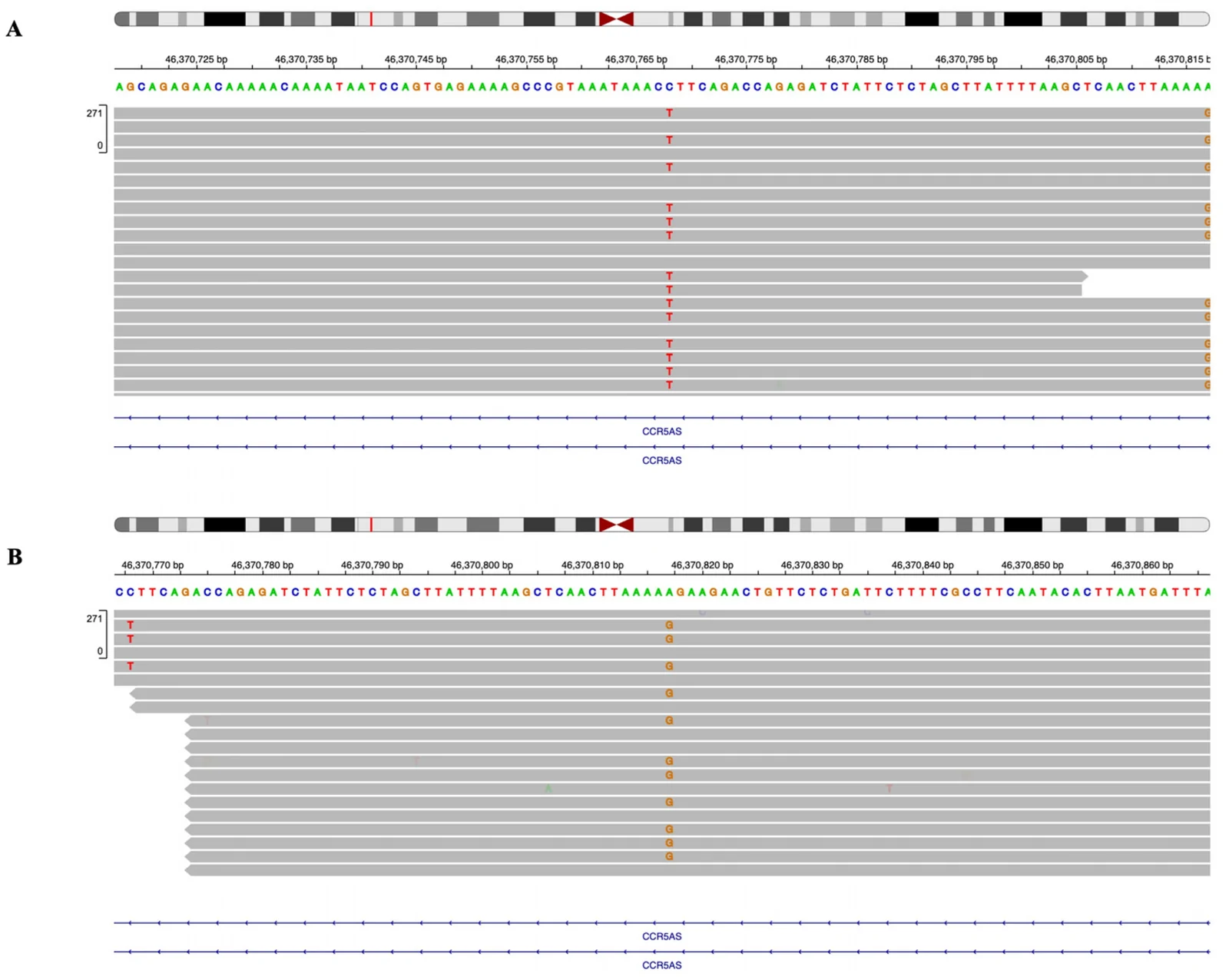
IGV visualization of the *CCR5* gene variants rs1799988:C > T (**A**) and rs1800023:A > G (**B**) identified by targeted sequencing.

According to the variant annotation in the sequencing pipeline, the *CCR5* variants rs1799988 and rs1800023 were classified as benign. Among the *CXCR4* variants, rs139877430 was classified as likely benign, while rs2228014:G > A, rs2471859:A > G, and rs2680880:A > T were classified as benign. No pathogenic or likely pathogenic variants were identified in the analyzed *CCR5* or *CXCR4* variants. Among the identified *CCR5* variants, rs1799988:C > T and rs1800023:A > G were selected for further analysis based on their relevance to the study objectives. The identified *CCR5* rs1799988:C > T and rs1800023:A > G variants were visualized using Integrative Genomics Viewer (IGV), as shown in Figure 1.

**Table 1:** Genetic variants identified in the *CCR5* and *CXCR4* genes in patients with epilepsy.

Patients with Epilepsy							
	Gene Variant	dbSNP ID	HGVS GRCh38-Positions	Variation Type	ACMG Classification	Amino Acid Change	Highest Population MAF
1	CCR5:c.-232C > T	rs1799988	chr3-46370768 C > T	Upstream gene variant	Benign	-	0.441
2	CCR5:c.-183A > G	rs1800023	chr3-46370817 A > G	Upstream gene variant	Benign	-	0.353
3	CCR5:c.554_585del	rs333	chr3-46373452 TACAGTCAGTATCAATTCTGGAAGAATTTCCAG > T	Frameshift variant	Benign	p.Ser185Ilefs*32	0.114
4	CXCR4:c.16-653C > T	rs139877430	chr2-136116565 G > A	Intron variant	Likely Benign	-	0.028
5	CXCR4:c.414C > T	rs2228014	chr2-136115514 G > A	Synonymous variant	Benign	p.Ile138=	0.035
6	CXCR4:c.16-522T > C	rs2471859	chr2-136116434 A > G	Intron variant	Benign	-	0.229
7	CXCR4:c.16-67T > A	rs2680880	chr2-136115979 A > T	Intron variant	Benign	-	0.589

dbSNP, Single Nucleotide Polymorphism database; HGVS, Human Genome Variation Society; MAF, minor allele frequency. Genomic positions are based on the GRCh38 reference genome. Variant classification was performed according to the ACMG/AMP guidelines. Population allele frequencies were obtained from the GnomAD database.

Although seven variants were identified by targeted sequencing, sufficient genotype variation for statistical comparison between the persistent and responsive groups was observed only for *CCR5* rs1799988:C > T and rs1800023:A > G. The remaining five variants (rs333, rs139877430:G > A, rs2228014:G > A, rs2471859:A > G, and rs2680880:A > T) showed low genotype distributions, with the variant genotypes observed in only a small number of individuals. Therefore, these variants were not included in the genotype-based statistical analyses because the low number of observations did not allow meaningful or reliable group-wise comparisons. Accordingly, HWE analyses were restricted to the two variants with sufficient genotype variation for the planned statistical analyses. The genotype distributions of the *CCR5* rs1799988:C > T and rs1800023:A > G variants were assessed for Hardy–Weinberg equilibrium (HWE) in the persistent and responsive groups. For rs1799988:C > T, the observed genotype frequencies were consistent with the expected frequencies in the persistent group (χ^2^ = 0.11, *p* = 0.738) and in the responsive group (χ^2^ = 0.06, *p* = 0.796). Similarly, the genotype distribution of rs1800023:A > G was in accordance with HWE in both the persistent (χ^2^ = 0.11, *p* = 0.738) and responsive (χ^2^ = 0.50, *p* = 0.405) groups (Table 2). Thus, neither variant showed a significant deviation from HWE in either seizure-control group.

No significant differences were observed in the genotype or allele distributions of *CCR5* rs1799988:C > T between the responsive and persistent groups. Similarly, no significant association was detected for rs1800023:A > G under genotypic, dominant, recessive, or allelic models (all *p* > 0.05).

### 2.2. CCR5 Haplotype and Linkage Disequilibrium Analysis

**Figure 2:**
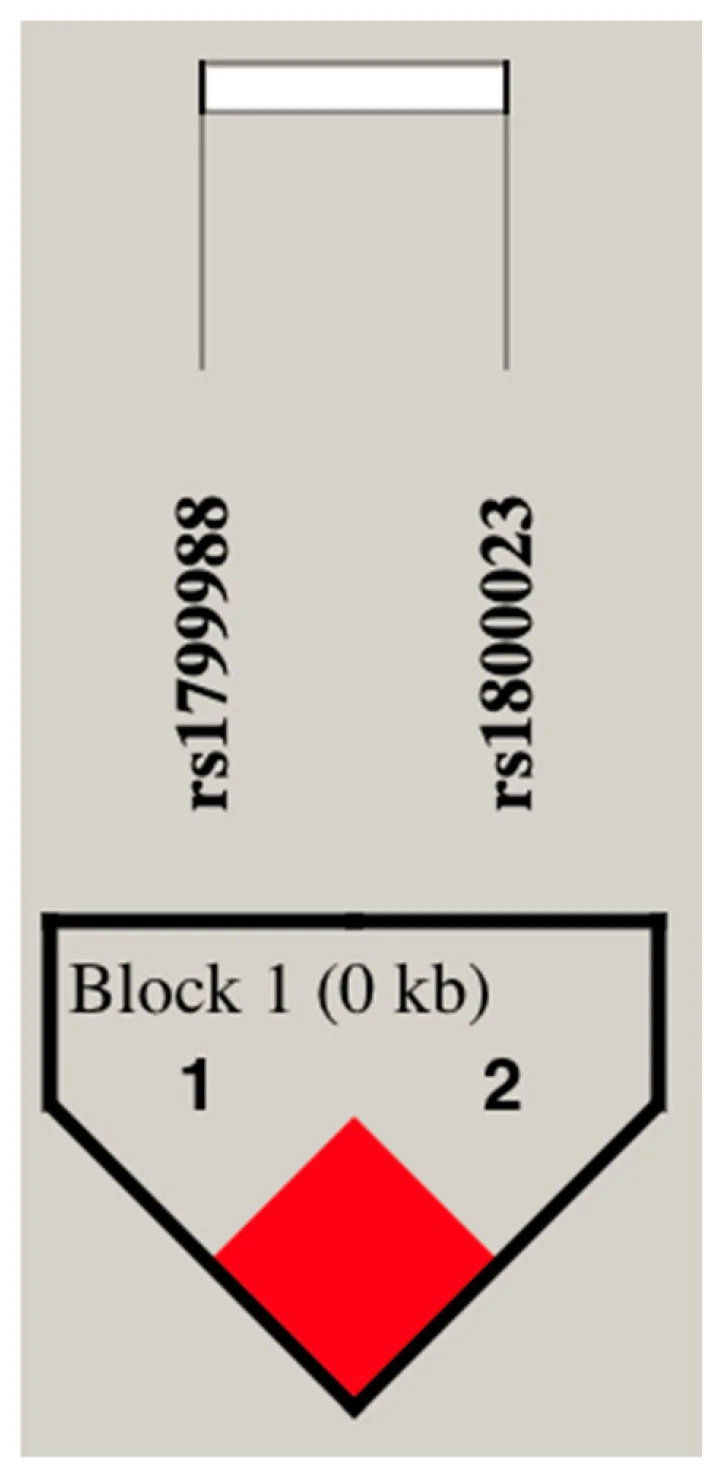
The *CCR5* gene rs1799988 and rs1800023 polymorphisms in a single haplotype block. Linkage disequilibrium: D′ = 1.000; r^2^ = 0.913.

Haplotype analysis was performed for the two *CCR5* polymorphisms, rs1799988:C > T and rs1800023:A > G (Table 3). The two variants showed strong linkage disequilibrium (LD), with a D′ value of 1.000 and an r^2^ value of 0.913 and were therefore included in a single haplotype block (Figure 2). Three haplotypes (TG, CA, and TA) were identified. The TG haplotype was the most frequent, with an overall frequency of 0.500, and showed identical frequencies in the responsive and persistent groups (0.500 vs. 0.500; χ^2^ = 0.000, *p* = 1.000). The CA haplotype was observed at frequencies of 0.500 and 0.462 in the responsive and persistent groups, respectively (χ^2^ = 0.063, *p* = 0.802). The rare TA haplotype was observed only in the persistent group (0.000 vs. 0.038); however, this difference was not statistically significant (χ^2^ = 0.708, *p* = 0.400). None of the *CCR5* haplotypes was significantly associated with seizure control (Table 4).

**Table 3:** *CCR5* Gene rs1799988 and rs1800023 Variations in Haplotype Analysis Markers.

Gene	Marker	Polymorphism	Allele Change	Contig Position
*CCR5*	Marker 1	rs1799988	C > T	46370768
	Marker 2	rs1800023	A > G	46370817

**Table 4:** Single and Block Haplotype Analysis of Variations in the *CCR5* Gene.

Frequencies						
Marker	Haplotype	Frequency	Responsive Group	Persistent Group	Chi Square	*p* Value
M1 + M2	TG	0.500	0.500	0.500	0.000	1.000
M1 + M2	CA	0.477	0.500	0.462	0.063	0.8017
M1 + M2	TA	0.023	0.000	0.038	0.708	0.400

## 3. Discussion

In this exploratory study, we characterized genetic variation in CCR5 and CXCR4 in 22 patients with epilepsy and examined its potential relationship with seizure-control status. Seven variants were identified; however, the low number of carriers for several variants limited the scope of genotype-based association analyses to CCR5 rs1799988:C > T and rs1800023:A > G. No statistically significant associations were detected between these variants and seizure-control status. The observed linkage disequilibrium between the two CCR5 variants was consistent with previously reported patterns and should be regarded as a descriptive finding rather than a novel contribution. Given the limited sample size, the present findings are insufficient to determine whether genetic variation in either gene is associated with seizure control.

CCR5 is a chemokine receptor involved in immune-cell recruitment and inflammatory signaling and has been implicated in neuroinflammatory processes within the central nervous system [8,13]. CCR5 signaling may contribute to the recruitment and activation of inflammatory cells and to the regulation of microglial responses following seizure activity [8]. Experimental studies have suggested that alterations in the CCL5/CCR5 axis may influence neuroinflammatory responses and neuronal injury in epilepsy models [9]. These biological properties make CCR5 a plausible candidate gene for investigating interindividual variation in epilepsy. However, the biological plausibility of CCR5 does not necessarily imply a direct relationship between *CCR5* genetic variation and treatment response, which requires specific clinical and genetic evaluation.

In the present exploratory study, no significant differences were observed in the genotype or allele distributions of *CCR5* rs1799988:C > T and rs1800023:A > G between the seizure-control and persistent-seizure groups. Both rs1799988:C > T and rs1800023:A > G are upstream regulatory region variants of *CCR5* that have previously been investigated in human populations in the context of immune-mediated and inflammatory conditions [14,15]. Likewise, neither dominant nor recessive genetic models revealed significant associations with seizure control, and no significant associations were observed for the *CCR5* haplotypes. Although no significant association was detected between these variants and seizure control, the strong linkage disequilibrium was observed between rs1799988:C > T and rs1800023:A > G (D′ = 1.000, r^2^ = 0.913). This finding suggests that these two variants are strongly correlated in the present cohort and may represent a linked genetic background rather than independent sources of genetic variation. Such linkage may be relevant when considering the combined contribution of *CCR5* variants in future studies, although the clinical significance of this finding remains to be established.

The potential functional relevance of the *CCR5* promoter variants identified in this study warrants consideration. Previous in silico analyses have suggested that *CCR5* promoter polymorphisms may influence transcription factor binding, with rs1799988 predicted to affect a glucocorticoid receptor alpha binding region [14]. In addition, previous studies have reported differences in *CCR5* promoter activity associated with promoter haplotypes [15]. However, the specific functional effects of the individual rs1799988:C > T and rs1800023:A > G variants have not been directly validated experimentally. Further functional studies are therefore needed to clarify their potential regulatory effects.

The *CCR5* rs333 (CCR5-Δ32) variant was identified in two heterozygous patients in our cohort. However, the limited number of carriers precluded a reliable assessment of its association with treatment response. Although the functional consequences of the CCR5-Δ32 variant have been well documented in previous studies, these established biological effects should not be interpreted as evidence of an association in the present cohort. Therefore, the identification of rs333 in two patients should be considered a descriptive finding, and larger studies are required to determine whether this variant has any relevance to seizure control.

Four genetic variants were identified in *CXCR4*, including intronic and synonymous variants. However, several of these variants were observed at low frequencies in the study cohort, limiting the ability to perform robust genotype-based comparisons and to draw meaningful conclusions regarding their potential relationship with seizure control. Although CXCR4 is biologically relevant to chemokine-mediated inflammatory signaling and has been implicated in neuroinflammatory processes associated with epilepsy [16], the present findings do not provide evidence for a significant association between the identified *CXCR4* variants and seizure control.

The observed variants may nevertheless provide a basis for further investigation of the contribution of chemokine receptor genetic variation to epilepsy and seizure control in larger cohorts. Chemokine receptors such as CCR5 and CXCR4 are involved in immune-cell trafficking and inflammatory signaling, and neuroinflammatory mechanisms have increasingly been implicated in epilepsy. Although a direct mechanistic link between genetic variation in these receptors and seizure control has not been established, alterations in chemokine signaling could potentially modify inflammatory processes that influence neuronal function and the biological response to seizure control. Thus, the potential relevance of *CCR5* and *CXCR4* genetic variation to treatment response should be considered a hypothesis requiring further investigation rather than an established mechanism.

Our previous studies in Turkish patients with epilepsy have also investigated genetic variation in ion-channel genes, including *SCN1A*, *CACNA2D2*, and voltage-gated potassium channel genes, in relation to antiseizure medication response [17,18]. These studies, together with the present findings, support the investigation of diverse biologically relevant genetic pathways as potential contributors to interindividual variability in epilepsy treatment outcomes. However, the potential role of chemokine receptor variation in seizure control remains to be established and requires validation in larger, adequately powered cohorts.

### Limitations

Several limitations should be considered when interpreting these findings. The small sample size substantially limited statistical power and the precision of association estimates. This limitation was particularly important for the five variants with low numbers of carriers, for which meaningful genotype-based comparisons could not be performed. Therefore, the absence of statistically significant associations should not be interpreted as evidence that these variants have no effect on seizure control.

Clinical heterogeneity, including differences in age, sex, disease characteristics, and antiseizure medication regimens, may have contributed to variability in seizure control. These potential confounding factors could not be adequately accounted for in multivariable analyses because of the limited sample size. Furthermore, although all participants were recruited from the same geographical region and study setting, ancestry-related population structure could not be formally assessed using ancestry-informative markers or genome-wide genetic data.

The observed linkage disequilibrium between CCR5 rs1799988 and rs1800023 is a descriptive finding consistent with previous reports and should not be interpreted as a novel finding or as evidence of an independent association with seizure control. The study also lacked functional validation and an independent replication cohort, limiting the ability to establish the biological relevance and reproducibility of the observed findings.

The findings should be considered preliminary and hypothesis-generating rather than definitive evidence of pharmacogenetic associations. Larger studies with adequate statistical power, well-characterized clinical phenotypes, standardized treatment exposure, appropriate adjustment for potential confounders, ancestry-related genetic data, and independent replication are needed to determine whether genetic variation in CCR5 or CXCR4 is associated with seizure control in epilepsy.

## 4. Materials and Methods

### 4.1. Study Design

In this study, 22 patients with epilepsy who had been receiving antiseizure medication (ASM) treatment for at least five years were evaluated. All participants received valproic acid and/or levetiracetam as monotherapy or polytherapy. Patients who had experienced three or more seizures during the previous year despite ongoing ASM treatment were classified as the persistent-seizure group. Patients who had remained seizure-free for more than one year while receiving ASM treatment were classified as the responsive group [19]. Thus, this classification was based on observed seizure frequency during the 12-month assessment period and was not intended to constitute a formal classification of drug-resistant epilepsy according to the ILAE definition. Seizure-control status was determined based on the patients’ clinical records and treatment history. Patients were not selected based on treatment response or resistance, and no specific seizure type was excluded. Both patients with adequate seizure control and those with persistent seizures during ASM therapy were included and subsequently classified according to their observed seizure-control status. The study was approved by the Haliç University Ethics Committee (Reference number 2025/355) and was conducted in accordance with the Declaration of Helsinki. Written informed consent was obtained from all participants. Peripheral whole-blood samples were obtained from the Republic of Turkey Ministry of Health Sinan Sipahi Family Health Center.

### 4.2. Next-Generation Sequencing Method

Genomic DNA was isolated from EDTA-coated peripheral blood samples using the Genomic DNA-Column Kits (QIAGEN^®^, Silicon Valley, CA, USA, cat. nos. 51304). NanoDrop 2000c (Thermo-Fisher Sci., Waltham, MA, USA) was used to check the purity and concentration of DNA samples. Firstly, the exons of *CCR5* and *CXCR4* genes and the regions located at the exon and exon-intron boundaries were amplified using a PCR device (C1000; Biorad, Hercules, CA, USA). Libraries were constructed using the TruSeqCustom Amplicon v1.5 Exome Library Prep Kit (Illumina, Inc., San Diego, CA, USA, cat, nos. 20018705), and all samples were sequenced on the NovaSeq 6000 System (Illumina, Inc., San Diego, CA, USA) platform.

### 4.3. Analyzing Raw Data

In previous studies, we achieved over 20-fold coverage for 95% of the targeted regions, as detailed in the methodology [20,21]. Sequencing data were initially evaluated for read quality with FastQC v0.11.9. Adapter sequences and low-quality bases were subsequently removed using Cutadapt v5.0. The resulting high-quality reads were mapped to the human GRCh38 reference genome and downstream read processing and variant calling were performed using the DRAGEN v4.3.6 pipeline. Functional and genomic annotations of the identified variants were generated with Ensembl Variant Effect Predictor (VEP) v115.2. Candidate variants were then inspected manually in the Integrative Genomics Viewer (IGV) v3.8.3 to confirm the sequencing. A minimum read depth of 30× was applied for variant assessment, and 95% coverage of the targeted regions was achieved. These quality control criteria were used to support the reliability of variant detection and subsequent analyses.

Variants with a minor allele frequency (MAF) below 1% in the gnomAD v4.1 database were considered rare and, where relevant, prioritized for further evaluation based on their potential functional effects. Population allele frequencies reported in Table 1 were obtained from the GnomAD v4.1 database. Variant interpretation was performed using the Ilyome Bioinformatics platform. The clinical significance of variants was assessed according to the 2015 standards and guidelines established by the American College of Medical Genetics and Genomics and the Association for Molecular Pathology (ACMG/AMP) [22].

### 4.4. Statistical Analyzes

Statistical analysis was carried out using GraphPad Prism version 10.3.1 software. The Chi-square test was applied to compare the study groups. Fisher’s exact test was utilized when any expected cell frequency was below 5. Allele and genotype frequencies were calculated, and Hardy–Weinberg equilibrium estimates were also determined. Deviations from Hardy–Weinberg equilibrium were analyzed using Michael H. Court’s (2005–2008) online calculator. Haplotype analysis was performed using the Haploview 4.1 software for the CCR5 rs1799988 and rs1800023 variants, which had sufficient genotype variation for haplotype estimation. The remaining five low-frequency variants were not included in the haplotype analysis because their low genotype frequencies did not allow reliable estimation of haplotype frequencies. A *p*-value of less than 0.05 was regarded as statistically significant.

No correction for multiple testing was applied, as none of the seven identified variants showed a nominally significant association with treatment response (all *p* > 0.05). Given the small sample size and the low number of carriers for several variants, the analyses were not intended to provide definitive evidence of association. Therefore, the association analyses were considered exploratory.

## 5. Conclusions

In conclusion, this exploratory study identified genetic variation in CCR5 and CXCR4 among patients with epilepsy but did not detect statistically significant associations between the investigated variants and seizure control during treatment. Given the small sample size and the low frequency of several variants, these findings should not be interpreted as evidence against a potential role of *CCR5* and *CXCR4* genetic variation in seizure control. Rather, they provide preliminary, hypothesis-generating observations that require validation in larger, adequately powered cohorts.

## Figures and Tables

**Table 2:** Hardy–Weinberg equilibrium between the groups for *CCR5* rs1799988:C > T, and *CCR5* rs1800023:A > G.

*CCR5* rs1799988:C > T					
Groups	Genotypes	Observed	Expected	χ^2^	*p*
Persistent	CC	2	2.3	0.11	0.738
	CT	5	4.5		
	TT	2	2.3		
Responsive	CC	3	2.8	0.06	0.796
	CT	6	6.5		
	TT	4	3.8		
***CCR5*** **rs1800023:A > G**					
**Groups**	**Genotypes**	**Observed**	**Expected**	**χ^2^**	***p***
Persistent	AA	2	2.3	0.11	0.738
	AG	5	4.5		
	GG	2	2.3		
Responsive	AA	4	3.3	0.50	0.405
	AG	5	6.5		
	GG	4	3.3		

## Data Availability

The original contributions presented in this study are included in the article. Further inquiries can be directed to the corresponding author.
